# CAG Repeat Variants in the POLG1 Gene Encoding mtDNA Polymerase-Gamma and Risk of Breast Cancer in African-American Women

**DOI:** 10.1371/journal.pone.0029548

**Published:** 2012-01-20

**Authors:** Sami Azrak, Vanniarajan Ayyasamy, Gary Zirpoli, Christine Ambrosone, Elisa V. Bandera, Dana H. Bovbjerg, Lina Jandorf, Gregory Ciupak, Warren Davis, Karen S. Pawlish, Ping Liang, Keshav Singh

**Affiliations:** 1 Department of Biological Sciences, Brock University, St. Catharine's, Ontario, Canada; 2 Department of Cancer Genetics, Roswell Park Cancer Institute, Buffalo, New York, United States of America; 3 Department of Cancer Prevention and Control, Roswell Park Cancer Institute, Buffalo, New York, United States of America; 4 The Cancer Institute of New Jersey, New Brunswick, New Jersey, United States of America; 5 University of Pittsburgh Cancer Institute, University of Pittsburgh, Pittsburgh, Pennsylvania, United States of America; 6 Department of Oncological Sciences, Mount Sinai School of Medicine, New York, New York, United States of America; 7 New Jersey State Cancer Registry, New Jersey Department of Health & Senior Services, Trenton, New Jersey, United States of America; 8 Departments of Genetics, Pathology, Environmental Health, Center for Free Radical Biology, Center for Aging and UAB Comprehensive Cancer Center, University of Alabama at Birmingham, Birmingham, Alabama, United States of America; University of Windsor, Canada

## Abstract

The DNA polymerase-gamma (*POLG*) gene, which encodes the catalytic subunit of enzyme responsible for directing mitochondrial DNA replication in humans, contains a polyglutamine tract encoded by CAG repeats of varying length. The length of the CAG repeat has been associated with the risk of testicular cancer, and other genomic variants that impact mitochondrial function have been linked to breast cancer risk in African-American (AA) women. We evaluated the potential role of germline *POLG*-CAG repeat variants in breast cancer risk in a sample of AA women (100 cases and 100 age-matched controls) who participated in the Women's Circle of Health Study, an ongoing multi-institutional, case-control study of breast cancer. Genotyping was done by fragment analysis in a blinded manner. Results from this small study suggest the possibility of an increased risk of breast cancer in women with minor CAG repeat variants of *POLG*, but no statistically significant differences in CAG repeat length were observed between cases and controls (multivariate-adjusted odds ratio 1.74; 95% CI, 0.49–6.21). Our study suggests that *POLG-*CAG repeat length is a potential risk factor for breast cancer that needs to be explored in larger population-based studies.

## Introduction

Despite the overall higher incidence of breast cancer in European-American (EA) women as compared to African-American (AA) women, AA women are more likely to be diagnosed with breast cancer before age 40 and to have tumors with aggressive pathological characteristics, including high tumor grade, lack of expression of estrogen and progesterone receptors (ER, PR) and HER2 (triple-negative breast cancers), and additional features of basal-like breast cancer (ER−/PR−/HER2−/cy5/6+/EGFR+) [1]. Triple-negative and basal-like breast cancers are recognized to be associated with a considerably poorer prognosis than other breast cancer subtypes [2]. Although the role of genetic factors, including polymorphisms in the BRCA1, BRCA2, ATM, CHEK2, p53, PTEN, NBS1, RAD50, BRIP1, and PALB2 genes have been extensively studied in breast cancer in EA populations [3], there are few studies of the role of common variants in nuclear gene(s) related to mitochondrial function in the etiology of breast cancer in AA women.

A recent study demonstrated that tumor-cell mitochondrial DNA copy (mtDNA) number correlates with tumor progression as well as patient prognosis and disease-free survival in breast cancer [4]. Cellular mtDNA copy number (or content) is controlled by the nuclear-encoded POLG gene encoding the only known mtDNA polymerase (polymerase-gamma) in humans. Our previous research suggests that mutations in the *POLG* gene may result in depletion of mtDNA and confer breast cancer phenotype [5]. Human *POLG* consists of an exonuclease domain with three exonuclease motifs, I, II and III, and a polymerase domain with three polymerase motifs, A, B and C, along with an intervening linker region [6]. The exonuclease domain of POLB, which is responsible for the proof-reading activity of the encoded enzyme, harbors a CAG repeat region in exon 2. The contraction of CAG repeats in *POLG* affects its expression [7], and an expanded CAG repeat sequence seems to confer toxic functions on the protein through protein-protein interactions [8]. The CAG repeat polymorphism has been studied in several diseases, including male infertility and neurodegeneration [9]–[11]. An association of the *POLG*-CAG repeat expansion with testicular cancer has also been reported [12]. Previous studies have also suggested that variation in the number of CAG repeats in androgen- and estrogen-receptor genes influence the risk of breast cancer [13]. However, the significance of the CAG repeat polymorphism of *POLG* in breast cancer has not been investigated. The purpose of this study was to evaluate the association of *POLG* CAG repeat length with breast cancer risk in the Women's Circle of Health Study (WCHS), an epidemiological case-control study of breast cancer in AA and EA women.

## Materials and Methods

### Study subjects

The data and samples from 100 AA women with breast cancer and 100 age-matched AA controls for this study were obtained from the Women's Circle of Health Study (WCHS), a case-control study specifically designed to investigate risk factors for early, aggressive breast cancer in AA women. The WCHS has been previously described [14]. Briefly, women with incident, primary breast cancer were identified through both hospital-based case ascertainment at hospitals in the New York City area and population-based case ascertainment in seven counties in northern New Jersey through the New Jersey State Cancer Registry, a National Cancer Institute Surveillance, Epidemiology, and End Results (SEER) registry. Controls without breast cancer were identified by random-digit dialing and matched to cases by telephone prefixes and 5-year age intervals. All study participants completed an in-person, structured interview to obtain information about lifestyle, reproductive, and medical histories, demographics and other variables; AA race was determined by self-report. Participants provided either a blood or saliva sample at enrollment. Body mass index (BMI) was assessed by measuring height and weight at the interview and was calculated as weight in kilograms (kg) divided by height in meters squared (m^2^). All study subjects provided written informed consent for the study and use of biospecimens, and the study was approved by the Institutional Review Boards at Mount Sinai School of Medicine (MSSM) (coordinating site for NY enrollment and NYC controls), the local NYC hospital (for cases), The Cancer Institute of New Jersey (coordinating site for NJ enrollments) as well as at Roswell Park Cancer Institute IRB approval number I-120807.

### DNA isolation and PCR

Genomic DNA was extracted from whole blood and evaluated for purity and concentration using a Nanodrop UV spectrophotometer and quantified on a spectrofluorometer (Gemini XS SPECTRAmax, Molecular Devices, Sunnyvale, CA, USA) using the PicoGreen dsDNA quantification kit, as per the manufacturer's instructions (Invitrogen, Carlsbad, CA, USA).. Double-stranded DNA was quantitated using a PicoGreen-based fluorometric assay. Primers (*POLG*_F: 6FAM-tggatgtccaatgggttgtgc and *POLG*_R: aagccaggtgttctgactcc) were designed to amplify a 275-bp fragment encompassing CAG repeats present in exon 2 of *POLG1*. The amplification was carried out in a 25 ul reaction containing approximately 50 ng of template DNA, 0.6 uM of both forward and reverse primers, and 0.5 ul AccuPrime™ *Taq* DNA polymerase (Invitrogen, USA) using a TECHNE TC-412 (96×0.2 ml) thermocycler (MIDSCI, USA). The PCR conditions were as follows: initial denaturation at 94°C for 4 min in 26 amplification cycles at 94°C for 10 sec, annealing at 61°C for 30 sec and 68°C for 40 sec, followed by a final extension step at 68°C for 5 min.

### Genotyping assays

Fluorescently labeled fragments generated by PCR were analyzed on ABI PRISM 3130XL (Applied biosystems, USA), according to the DNA fragment analysis protocol. DNA fragment data were collected and then visualized using GeneMapper® software. The data containing the genomic location (peak size) for all samples were exported as delimited text files and blindly analyzed for the number of CAG repeats in each sample. Assay reproducibility was tested using multiple runs of both positive and negative samples with different CAG repeat variants.

### Statistical Analysis

Differences between categorical variables were assessed with the chi-square test, and continuous variables were compared using Student's t-test. All statistical tests were two-sided. Age at menarche and the number of children were divided into tertiles using cut-points based on the distribution of these variables among controls. Age at first pregnancy was categorized as nulliparous, ≤19, 20–24, 25–29, or ≥30. Menopausal status at the time of cancer diagnosis was determined during the interview and categorized as premenopausal, perimenopausal, or postmenopausal. Crude and multivariate unconditional logistic regression models adjusted for age, age at menarche, age at first full-term pregnancy, menopausal status (menopausal, perimenopausal, postmenopausal), first-degree family history of breast cancer (yes/no) and BMI were used to calculate odds ratios (OR) and corresponding 95% confidence intervals (CI) as a measure of association between CAG repeats of mitochondrial DNA *POLG* and breast cancer risk. All statistical analyses were computed with SAS 9.2 (SAS Institute, Cary, NC, USA).

## Results

**Figure 1:**
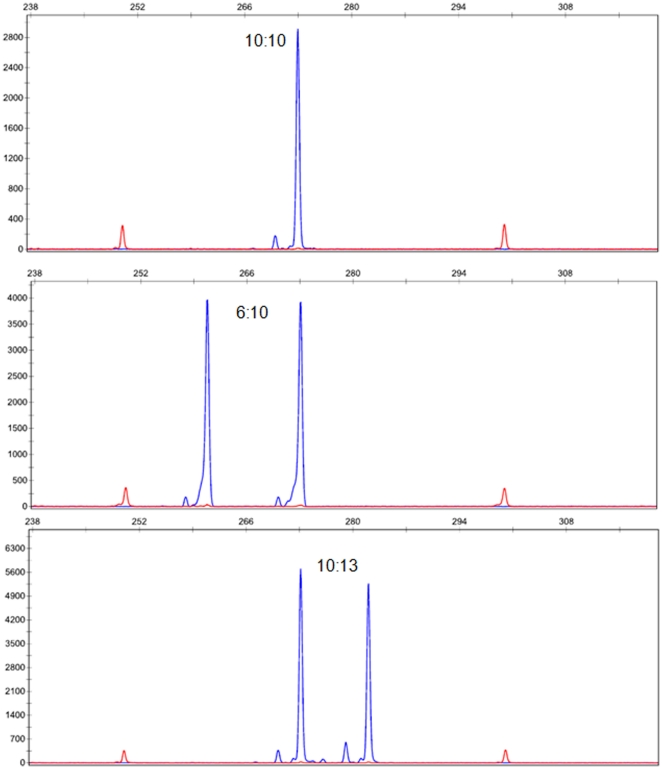
Representative electopherogram of normal CAG (10∶10), contraction (6∶10) and expansion (10∶13) of POLG.

The CAG fragment analysis was done in a blinded manner and repeat length was carefully assigned based on the peak size. After the initial observation, we eliminated 12 samples because they showed poor signal due to inadequate DNA. In this subset of the WCHS, there were no statistically significant differences between cases and controls with respect to age at menarche, age at first pregnancy, number of children, menopausal status, and family history of breast cancer (Table 1). We identified 7 breast cancer cases (7%) and 6 controls (6%) in which the common 10/10 *POLG*-CAG repeat was absent. The common 10/10 CAG repeat occurred in 52 cases (55%) and 56 controls (60%); heterozygous 10/non-10 CAG repeats were observed in 36 cases (38%) and 31 controls (33%) (Table 2). The representative CAG repeat contraction and expansion variants are shown as electropherograms (Figure 1).

**Table 1:** Selected characteristics of Cases and Controls in WCHS with POLG-CAG repeat polymorphism.

Characteristic	Cases (n = 95)n (%)	Controls (n = 93)n (%)	p^1^
POLG			0.75
10/10	52 (54.7)	56 (60.2)	
10/not 10	36 (37.9)	31 (33.3)	
Not 10/not 10	7 (7.4)	6 (6.5)	
Age at menarche			0.68
≤12	50 (53.2)	44 (47.3)	
12.5–13	20 (21.3)	24 (25.8)	
13.5+	24 (25.5)	25 (26.9)	
Age at first pregnancy			0.95
Nulliparous	20 (21.1)	22 (26.7)	
≤19	23 (24.2)	19 (20.4)	
20–24	26 (27.4)	25 (26.9)	
25–29	16 (16.8)	15 (16.1)	
30+	10 (10.5)	12 (12.9)	
Number of children			0.86
0–1	42 (44.2)	42 (45.2)	
2–3	37 (39.0)	38 (40.9)	
4+	16 (16.8)	13 (14.0)	
Menopausal Status			0.84
Premenopausal	35 (42.2)	34 (39.1)	
Perimenopausal	19 (22.9)	23 (26.4)	
Postmenopausal	31 (36.5)	31 (35.2)	
First degree relative with breast cancer			0.57
No	80 (84.2)	81 (87.1)	
Yes	15 (15.8)	12 (12.9)	

	Mean (Min-Max)	Mean (Min-Max)	p^2^
Age	50.4 (29–65)	50.0 (30–65)	0.78
BMI	31.4 (17.9–50.4)	31.6 (19.7–66.7)	0.85

1 Chi-square test.

2 t-test.

**Table 2:** Odd ratios of POLG-CAG repeat polymorphism in WCHS.

	Crude OR(95% CI)	AdjustedOR^1^ (95% CI)
POLG		
10/10	1.0 (ref)	1.0 (ref)
10/not 10	1.3 (0.68–2.30)	1.5 (0.73–2.96)
Not 10/not 10	1.3 (0.40–3.98)	1.7 (0.41–7.46)

1 Adjusted for age, age at menarche, age at first full term pregnancy, menopausal status, first degree family history of breast cancer, and BMI.

Compared with 10/10 repeats, the crude odds ratios (ORs) for 10/11, 10/12 repeats, and all other repeats were 1.16 (95% CI, 0.58–2.32), 0.93 (95% CI, 0.36–2.35), and 1.31 (95% CI, 0.46–3.76), respectively (Table 1). After adjustment for age, age at menarche, age at first full-term pregnancy, menopausal status, first-degree family history of breast cancer, and BMI, the adjusted ORs for 10/11 and 10/12 repeats were 1.15 (95% CI, 0.50–2.63) and 1.25 (95% CI, 0.45–3.46), respectively. In the case of CAG repeats other than 10/11 or 10/12, point estimates suggested an increased risk of breast cancer, but no statistically significant difference was observed in the frequency of CAG repeats between cases and controls (OR 1.74; 95% CI, 0.49–6.21, Table 2).

## Discussion

Mitochondrial *POLG* is the only polymerase known to be involved in human mitochondrial DNA replication. *POLG* contains CAG trinucleotide repeats in the coding region, and CAG repeat sequences are described to be highly unstable, leading to expansion or contraction of the repeat sequences [8]. In a recent study of the *POLG* gene, we reported the CAG repeat expansion occurs in 20% of breast cancer patients [5]. This pilot study suggests the possibility of an association between altered *POLG*-CAG repeat-length and an increased risk of breast cancer in AA women.

The presence of expanded or contracted CAG repeats has been linked with several diseases, including the human polyglutamine diseases [8]. Other epidemiologic studies have shown either a positive or negative correlation of CAG repeats with sporadic breast cancer, and one study reported an inverse correlation between *POLG*-CAG repeat length (r = −0.81) and the age of onset of disease in Friedreich's Ataxia (FRDA) patients [13]. In another study dealing with myotonic dystrophy, no significant difference in the frequency of CAG repeats was seen between cases and controls [7]. Studies of CAG-repeat polymorphisms in antiretroviral therapy-associated peripheral neuropathy similarly showed no significant associations [15]. However, CAG repeat-length polymorphisms have been associated with male infertility; the common 10-repeat variant of the CAG repeat was found to be significantly more prevalent among men with oligospermia [9] and unexplained subfertility [16].

There are two additional reports associating *POLG*-CAG repeat variants with cancer risk. Interestingly, a study of CAG repeat polymorphisms in the *POLG* gene in testicular cancer showed 36 (74%) wild-type homozygotes and 13 (26%) lacked one or both wild-type alleles, with the 10/11 variant in 10 patients and the 10/12, 10/6 and 11/11 variants in one patient each, suggesting that variants of the DNA POLG1 gene were more frequent in testicular cancer patients than in healthy men [12]. Another study suggested that the CAG polymorphism in *POLG* may be a contributing factor in the pathogenesis of testicular seminoma [*17*]. It should be emphasized, however, that existing studies in of *POLG*-CAG repeat variants in cancer compared the frequency of these variants in tumor tissue to the frequency in either surrounding normal tissue or blood samples. In contrast, our study evaluated germline polymorphisms in *POLG* in both breast cancer cases and in controls. Results of studies may differ depending on the DNA source due to additional clonal mutations that can occur in tumor cells.

This study is the first to investigate the role of CAG repeat polymorphisms in the nuclear *POLG* gene, which encodes mitochondrial DNA polymerase-gamma, in breast cancer risk. While our findings suggest a possible correlation between CAG repeat length and risk of breast cancer in AA women, the size of this genetic study sample was small, as evidenced by reduced power to demonstrate significant associations of known breast cancer risk factors such as age at menarche and family history of breast cancer with cancer risk in this study. Larger, preferably population-based, studies are needed in order to draw any firm conclusions about the role of *POLG*-CAG variants in breast cancer risk in AA or other populations.
